# The need of a weight management control program in judo: a proposal based on the successful case of wrestling

**DOI:** 10.1186/1550-2783-7-15

**Published:** 2010-05-04

**Authors:** Guilherme G Artioli, Emerson Franchini, Humberto Nicastro, Stanislaw Sterkowicz, Marina Y Solis, Antonio H Lancha

**Affiliations:** 1 Laboratory of Applied Nutrition and Metabolism. School of Physical Education and Sport. University of Sao Paulo, Brazil; 2 Martial Arts and Combat Sport Research Group. School of Physical Education and Sport. University of Sao Paulo, Brazil; 3 University School of Physical Education in Krakow, Poland

## Abstract

Judo competitions are divided into weight classes. However, most athletes reduce their body weight in a few days before competition in order to obtain a competitive advantage over lighter opponents. To achieve fast weight reduction, athletes use a number of aggressive nutritional strategies so many of them place themselves at a high health-injury risk. In collegiate wrestling, a similar problem has been observed and three wrestlers died in 1997 due to rapid weight loss regimes. After these deaths, the National Collegiate Athletic Association had implemented a successful weight management program which was proven to improve weight management behavior. No similar program has ever been discussed by judo federations even though judo competitors present a comparable inappropriate pattern of weight control. In view of this, the basis for a weight control program is provided in this manuscript, as follows: competition should begin within 1 hour after weigh-in, at the latest; each athlete is allowed to be weighed-in only once; rapid weight loss as well as artificial rehydration (i.e., saline infusion) methods are prohibited during the entire competition day; athletes should pass the hydration test to get their weigh-in validated; an individual minimum competitive weight (male athletes competing at no less than 7% and females at no less than 12% of body fat) should be determined at the beginning of each season; athletes are not allowed to compete in any weight class that requires weight reductions greater than 1.5% of body weight per week. In parallel, educational programs should aim at increasing the athletes', coaches' and parents' awareness about the risks of aggressive nutritional strategies as well as healthier ways to properly manage body weight.

## Introduction

Judo is an Olympic sport practiced all over the world. Some studies reported that judo athletes present highly developed strength, anaerobic power and capacity, aerobic power, flexibility and low levels of body fat [1]. A more detailed comparison between elite and non-elite judo competitors revealed that high level judo athletes present more developed upper body muscle mass, higher upper body anaerobic power and capacity, and higher ability to perform high-intensity intermittent specific judo activities [2]. In all official competitions, judo athletes are paired with opponents of similar body weight through weight classes. The aim of such division is to ensure fairness and promote evenhanded combats in terms of strength, leverage and agility. However, it is well known that most judo competitors use several harmful methods of rapid weight loss in an attempt to classify for a lighter weight class and, by doing so, to obtain competitive advantage against lighter and weaker opponents [3].

The rapid weight loss is a well documented problem in collegiate wrestling. Since the 1970's, studies have characterized the patterns of rapid weight loss among wrestlers [4,5]. Surveys addressing such patterns reported that ~80% of competitors engage in weight loss procedures [4,5]. According to these studies, the most prevalent nutritional strategies for reducing weight are severe fluid and food restriction, using saunas and heated rooms and exercising with rubberized suits. The use of diuretics, laxatives, diet pills and even self-induced vomiting are extreme methods often reported in the literature [4]. Athletes reduce body weight several times per season and the magnitude of weight cycling is of about 5% to 10% of body weight [4]. Athletes start losing weight very early in their competitive life. Although adolescence is the period during which athletes most often begin cutting weight, a few athletes might start unhealthy weight loss procedures at very early ages, as was the impressive case of a 5-year- old boy who fasted and restricted food ingestion under his father's advice [6]. Although much less attention has been given to judo, recent studies have shown that the patterns of rapid weight loss in judo are very similar and comparable to those reported in wrestling [3].

Rapid weight loss has been proven to negatively affect a number of health-related parameters. Briefly, it can lead to acute cardiovascular dysfunctions [7], immunosuppression [8], lowered bone density [9], impaired thermoregulation [10], impaired cognitive function [11], negative mood state [12], hormonal unbalance [13], temporary growth impairment [14], poor nutritional status [15], increased injury risk [16] and increased risk of developing eating disorders [4,17]. Although some studies have demonstrated that rapid weight loss impairs high-intensity performance [18-20], no negative effects have been observed [21,22] if athletes are allowed to recovery for at least 3-4 hours from weight loss (i.e., they are allowed to eat and drink as much as they want before the performance tests take place). Of note, in virtually all judo competitions, each first match begins within an average of ~3-6 hours after the weigh-in and this duration frequently lasts longer. Together, these facts probably reinforce the competitive advantages of reducing the athletes' weight rapidly before competitions, encouraging them to engage in harmful weight management procedures.

Despite three official warnings from American College of Sports Medicine and American Medical Association [10,23,24], nothing had been done in order to prevent health injuries in consequence of rapid weight loss until the occurrence of three deaths of young wrestlers in the 1997 season. The deaths were associated to hyperthermia, which was probably caused by hypohydration as they were preparing for a competition and engaging in rapid weight loss regimens [25]. These athletes were reducing 15% of their body weight, on the average [26]. Only after these tragic events, the National Collegiate Athletic Association (NCAA) implemented a program for controlling the weight cutting, which was demonstrated to be efficient in reducing the prevalence of rapid weight loss among wrestlers and in attenuating the aggressiveness of the weight management behaviors [27].

In March 1996, the South Korean judo medalist Chung Se-hoon died of a heart attack probably triggered by an extreme rapid weight loss regime, because he was preparing for the 1996 Atlanta Olympic Games. However, the International Judo Federation has never considered the implementation of an official program aiming to discourage athletes from engaging in harmful weight loss procedures and, at present, the patterns of rapid weight loss among judo competitors are as inappropriate as those reported regarding wrestlers before the NCAA's weight control program [3]. Hence, it is clear that a great number of judo athletes is in risk of health injuries and a weight control program for judo urgently needs to be created. Moreover, the interesting study of Alderman et al. [28] showed that the wrestlers who improved their weight management behaviors in scholastic wrestling (under the NCAA regulation) had an aggressive behavior when reducing weight for international style wrestling, which has no regulation regarding weight control. This clearly demonstrates that the most effective way to prevent athletes from reducing weight harmfully is through the use of strict regulations. Therefore, the purpose of the present manuscript is to highlight the necessity of a weight control program for judo and to propose the creation of new rules based on the successful program by NCAA for improving weight management behaviors.

## Discussion

The rules aiming to control weight cutting should be implemented by the International Judo Federation (IJF) and adopted by all National and Regional Federations in order to reach the highest possible impact and effectiveness. Obviously, this manuscript does not intend to present a final solution to the problem. Instead, we believe that this proposal must be discussed in light of the well-being and safety of the competitors and considering what is feasible in the competitive atmosphere before being implemented.

As previously mentioned, in almost all judo competitions, there is a relatively long period between the weigh-in and the first combat. Data from our group indicate that the interval normally lasts 3-6 h [29], but it can eventually last longer, especially when the weigh-in takes place on the day preceding the competition. Although physical performance is impaired after rapid weight loss [18-20], the interval of ~3-6 h allows the athletes to return several physiological variables close to baseline [7,30] and, most importantly, high-intensity anaerobic performance is also completely recovered [21,22]. Thus, it is likely that rapid weight loss will be attenuated by reducing this interval to 1 h, at the longest, because the athletes will feel the negative effects of weight loss on performance. After the weigh-in, some athletes can also use artificial rehydration methods, such as intravenous infusion of saline solution which is a time-demanding procedure. Reducing the time period between weigh-in and competition would also help athletes to avoid using such a procedure. Therefore, the first change in the rules proposed is to reduce the time interval between weigh-in and the first match to 1 h or less.

During the official weigh-in, athletes are allowed to be weighed-in as many times as needed. It means that an athlete whose weight is above the weight class limit is allowed to leave the weighing room, reduce the weight very quickly and return for a new weigh-in attempt. This can be repeated several times until the athlete reaches the desired weight, as long as the weigh-in period is not expired. To achieve this quick weight loss, athletes frequently exercise wearing vapor-impermeable suits under winter garments; also, they frequently spit or even induce vomiting. After the weigh-in, some athletes can also use artificial rehydration methods, such as intravenous infusion of saline solution. In view of this, the second and the third additional rules that should be considered for implementation are: allowing the athletes to weigh-in only once and to prohibit the use of any method of dehydration before the weigh-in and the use of any artificial rehydration method after the weigh-in. Moreover, penalizations to the athlete who is caught using such dehydrating or rehydrating methods should also be considered.

To avoid an athlete's weighing-in in a dehydrated state, hydration status should be assessed by using simple tests before or during weigh-ins. The technique for measuring hydration status has to be chosen based on the costs, portability, easiness of use and safety. Likewise, the level of compliance required from the athletes as well as the time and the technical expertise required from the competition's staff should also be considered. In this context, the techniques that best fit within these characteristics are urine color and urine specific gravity [31]. Urine specific gravity may be adequately used for determining hydration status, refractometry (a simple, fast and inexpensive technique) being the most reliable manner to assess specific gravity [32]. Although urine color test, which is even less expensive than specific gravity, would also be suitable for this purpose, this test is very subjective and subjected to errors due to visual misinterpretations. This indeed makes the urine specific gravity determined by a calibrated refractometer the preferred method for hydration level determination. No athlete failing the hydration test should be allowed to compete. Also, penalizations to a severely dehydrated athlete should be considered.

To determine an individualized minimum competitive weight would indeed dramatically reduce the prevalence and magnitude of rapid weight loss as well as the aggressiveness of the weight reduction methods used by athletes. In the NCAA weight certification program, every athlete has to be assessed for minimum weight at the beginning of the season; the minimum weight would be used to evaluate the weight classes in which the athlete would be able to compete along the season. Of note, a judo season normally comprises the whole competitive year. According to the new World Ranking, which was proposed by IJF for Olympic Games qualification and for identifying the leading athletes in each Olympic weight category, points are accumulated during the international competitions held between May 1^st ^of each year and April 30^th ^of the next year. This could be used as reference for a judo season.

The minimum weight is determined based on the pre-season body fat and body weight, both assessed in euhydrated state, which is confirmed through a hydration test. The minimum weight is considered as the lightest weight class in which an athlete would compete without lowering his body fat to less than 7%. Due to the differences in body composition, physiology and metabolism between men and women, the lowest limit of fat percentage for women athletes should be 12% instead of 7%. However, exceptions could apply for athletes presenting pre-season body fat lower than the 7% or 12% limit in an euhydrated state. In these cases, the minimum weight should be considered the current body fat as the lowest limit.

After the determination of the minimum weight, the athletes are not allowed to compete in a given weight class if the calendar requires losses greater than 1.5% of the body weight per week. In order to exemplify how to determine whether an athlete is or is not eligible for competing in a given tournament, an athlete weighing 66 kg and intending to compete at under 60 kg weight class will be hypothesized. If reducing to 60 kg does not imply reducing body fat to less than 7%, this athlete would be allowed to compete in the under 60-kg category only 7 weeks after the assessment (i.e., he needs to reduce 10% of initial body weight, which would take 7 weeks to be achieved if the maximum of 1.5% per week is followed). In the meantime, this athlete would be allowed to compete in a heavier weight class (e.g., 60-66 kg). Moreover, judo federations should create a structure for routinely assessing athletes' body weight and body composition because a constant follow-up would be certainly more effective in avoiding dramatic weight fluctuations. In fact, the more frequent the assessments, the better controlled the weight fluctuations would be. The exact time period between assessments has to be determined in light of local specificities and feasibility. However, one evaluation every six months seems to be reasonable and easy to be implemented.

Although many other specific regulations regarding the minimum weight exist in the NCAA program, the two main ideas (i.e., the preseason determination of a reliable minimum competitive weight and reductions no greater than 1.5% per week) should be used to create a similar group of rules for judo.

An important aspect of the weight management among judo competitors is that the earlier the athletes begin reducing their weight, the more extreme and aggressive their behavior tends to be [3]. In fact, judo athletes have been shown to start reducing weight at very early ages in their competitive lives (12 ± 6 years of age) [3]. In view of this, it is reasonable to affirm that young athletes are likely to be the weight management programs' most important targets. This is particularly relevant in the current competitive scenario in judo because the IJF has promoted the World Judo Championship for Juvenile athletes in 2009 and the Youth Olympic Games will occur in 2010.

## Conclusion

**Figure 1:**
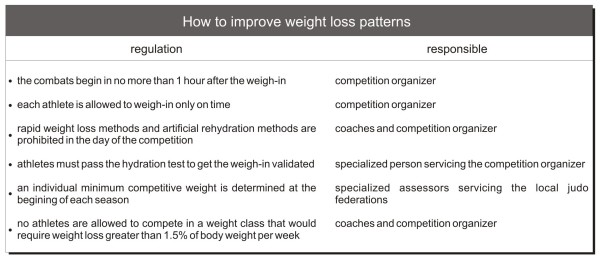
**Basic regulations to improve weight management behaviors among judo competitors**.

In conclusion, we propose six simple rules (Figure 1) that would probably improve the weight loss patterns among judo competitors. In parallel, International, National and Regional Judo Federations should establish educational programs for coaches, trainers, parents and athletes in order to increase awareness regarding the risks of extreme weight loss and healthier ways to manage body weight. This would also be of great importance for preventing judo athletes from failing in anti-doping tests because the program could decrease the use of diuretics. Together, the rules and the educational program would certainly improve the fairness of the game, making judo a safe, healthy and enjoyable sport.

## List of Abbreviations

NCAA: National Collegiate Athletic Association; IJF: International Judo Federation.

## Competing interests

The authors declare that they have no competing interests.

## Authors' contributions

GGA, HN, EF, SS, MYS and AHLJr have conceived the idea of the manuscript and established the manuscript's general structure. GGA has written the first draft and the other authors have equally contributed to the final version, which was approved by all authors.

## References

[B1] Thomas SG Cox MH LeGal YM Physiological profiles of the Canadian National Judo Team Can J Sport Sci 1989 14 142 147 2819609

[B2] Franchini E Takito MY Kiss MAPDM Physical fitness and anthropometrical differences between elite and non-elite judo players Biology of Sport 2005 22 315 328

[B3] Artioli GG Gualano B Franchini E Prevalence, magnitude, and methods of rapid weight loss among judo competitors Med Sci Sports Exerc 42 436 442 19952804 10.1249/MSS.0b013e3181ba8055

[B4] Steen SN Brownell KD Patterns of weight loss and regain in wrestlers: has the tradition changed? Med Sci Sports Exerc 1990 22 762 768 2287253 10.1249/00005768-199012000-00005

[B5] Tipton CM Tcheng TK Iowa wrestling study. Weight loss in high school students Jama 1970 214 1269 1274 10.1001/jama.214.7.1269 5536310

[B6] Sansone RA Sawyer R Weight loss pressure on a 5 year old wrestler Br J Sports Med 2005 39 e2 10.1136/bjsm.2004.013136 15618326 PMC1725024

[B7] Allen TE Smith DP Miller DK Hemodynamic response to submaximal exercise after dehydration and rehydration in high school wrestlers Med Sci Sports 1977 9 159 163 593078

[B8] Kowatari K Umeda T Shimoyama T Exercise training and energy restriction decrease neutrophil phagocytic activity in judoists Med Sci Sports Exerc 2001 33 519 524 11283425 10.1097/00005768-200104000-00003

[B9] Prouteau S Pelle A Collomp K Bone density in elite judoists and effects of weight cycling on bone metabolic balance Med Sci Sports Exerc 2006 38 694 700 10.1249/01.mss.0000210207.55941.fb 16679985

[B10] Oppliger RA Case HS Horswill CA American College of Sports Medicine position stand. Weight loss in wrestlers Med Sci Sports Exerc 1996 28 ix xii 8926865

[B11] Choma CW Sforzo GA Keller BA Impact of rapid weight loss on cognitive function in collegiate wrestlers Med Sci Sports Exerc 1998 30 746 749 10.1097/00005768-199805000-00016 9588618

[B12] Degoutte F Jouanel P Begue RJ Food restriction, performance, biochemical, psychological, and endocrine changes in judo athletes Int J Sports Med 2006 27 9 18 10.1055/s-2005-837505 16388436

[B13] Roemmich JN Sinning WE Weight loss and wrestling training: effects on growth-related hormones J Appl Physiol 1997 82 1760 1764 9173938 10.1152/jappl.1997.82.6.1760

[B14] Roemmich JN Sinning WE Weight loss and wrestling training: effects on nutrition, growth, maturation, body composition, and strength J Appl Physiol 1997 82 1751 1759 9173937 10.1152/jappl.1997.82.6.1751

[B15] Horswill CA Park SH Roemmich JN Changes in the protein nutritional status of adolescent wrestlers Med Sci Sports Exerc 1990 22 599 604 10.1249/00005768-199010000-00010 2233198

[B16] Green CM Petrou MJ Fogarty-Hover ML Injuries among judokas during competition Scand J Med Sci Sports 2007 17 205 210 17501865 10.1111/j.1600-0838.2006.00552.x

[B17] Oppliger RA Landry GL Foster SW Bulimic behaviors among interscholastic wrestlers: a statewide survey Pediatrics 1993 91 826 831 8464675

[B18] Fogelholm GM Koskinen R Laakso J Gradual and rapid weight loss: effects on nutrition and performance in male athletes Med Sci Sports Exerc 1993 25 371 377 8455453

[B19] Hickner RC Horswill CA Welker JM Test development for the study of physical performance in wrestlers following weight loss Int J Sports Med 1991 12 557 562 10.1055/s-2007-1024733 1797697

[B20] Horswill CA Hickner RC Scott JR Weight loss, dietary carbohydrate modifications, and high intensity, physical performance Med Sci Sports Exerc 1990 22 470 476 2402206

[B21] Artioli GG Iglesias RT Franchini E Rapid weight loss followed by recovery time does not affect judo-related performance J Sports Sci 2010 23 1 12 10.1080/02640410903428574 20035492

[B22] Klinzing JE Karpowicz W The effects of rapid weight loss and rehydratation on a wrestling performance test J Sports Med Phys Fitness 1986 26 149 156 3747480

[B23] ACSM American College of Sports Medicine position stand on weight loss in wrestlers Med Sci Sports 1976 8 xi xiii 957926

[B24] AMA Wrestling and weight control Jama 1967 201 131 133 10.1001/jama.201.1.131 6071692

[B25] Hyperthermia and dehydration-related deaths associated with intentional rapid weight loss in three collegiate wrestlers--North Carolina, Wisconsin, and Michigan, November-December 1997 MMWR Morb Mortal Wkly Rep 1998 47 105 108 9480411

[B26] Ransone J Hughes B Body-Weight Fluctuation in Collegiate Wrestlers: Implications of the National Collegiate Athletic Association Weight-Certification Program J Athl Train 2004 39 162 165 15173868 PMC419511

[B27] Oppliger RA Landry GL Foster SW Wisconsin minimum weight program reduces weight-cutting practices of high school wrestlers Clin J Sport Med 1998 8 26 31 10.1097/00042752-199801000-00007 9448954

[B28] Alderman BL Landers DM Carlson J Factors related to rapid weight loss practices among international-style wrestlers Med Sci Sports Exerc 2004 36 249 252 10.1249/01.MSS.0000113668.03443.66 14767247

[B29] Artioli GG Kashiwagura DB Fuchs MGC Recovery time after weigh-in during regional level judo championships. Annals of V IJF Judo Conference. Rio de Janeiro: International Judo Federation; 2007 (CD-Rom) 2007

[B30] Rankin JW Ocel JV Craft LL Effect of weight loss and refeeding diet composition on anaerobic performance in wrestlers Med Sci Sports Exerc 1996 28 1292 1299 8897387 10.1097/00005768-199610000-00013

[B31] Armstrong LE Assessing hydration status: the elusive gold standard J Am Coll Nutr 2007 26 575S 584S 17921468 10.1080/07315724.2007.10719661

[B32] Stuempfle KJ Drury DG Comparison of 3 Methods to Assess Urine Specific Gravity in Collegiate Wrestlers J Athl Train 2003 38 315 319 14737213 PMC314390

